# Comparisons of Periodontal Status between Females Referenced for Fertility Treatment and Fertile Counterparts: A Pilot Case–Control Study

**DOI:** 10.3390/ijerph17155281

**Published:** 2020-07-22

**Authors:** Vanessa Machado, João Botelho, Luís Proença, José João Mendes

**Affiliations:** 1 Periodontology Department, Clinical Research Unit (CRU), Centro de Investigação Interdisciplinar Egas Moniz (CiiEM), Instituto Universitário Egas Moniz (IUEM), 2829-511 Almada, Portugal; jbotelho@egasmoniz.edu.pt; 2 Clinical Research Unit (CRU), CiiEM, IUEM, 2829-511 Almada, Portugal; jmendes@egasmoniz.edu.pt; 3 Quantitative Methods for Health Research (MQIS), CiiEM, IUEM, 2829-511 Almada, Portugal; lproenca@egasmoniz.edu.pt

**Keywords:** female infertility, periodontitis, periodontal disease, fertility, women

## Abstract

Studies investigating the periodontal status of women seeking fertility treatment have never been conducted. The purpose of this pilot study was to compare the periodontal status among females referenced to a Fertility Clinic (FC) when compared to matched females from a representative regional epidemiological sample. Our secondary aims were to investigate if periodontal clinical measures differ between these two groups of females and how they impact on oral health-related quality of life (OHRQoL). We enrolled 18 women from an FC and 18 age, race and body mass index matched controls from the epidemiological survey Study of Periodontal Health in Almada-Seixal (SoPHiAS). In each subject, we performed a circumferential periodontal inspection to infer the periodontal status and applied a questionnaire measuring OHRQoL. FC females presented higher levels of periodontal disease, with higher periodontal epithelial surface area, periodontal probing depth and clinical attachment loss. However, periodontal diseases did not impact OHRQoL in this particular group of women seeking fertility care, suggesting unawareness about periodontal diseases. Within the limitations of this study, females referenced for fertility treatment presented worse periodontal measures than females from a representative control sample. These preliminary results may support future prospective studies to further explore the periodontal status and possible consequences in women seeking fertility care.

## 1. Introduction

According to the International Committee for Monitoring Assisted Reproductive Technology (ICMART) and the World Health Organization (WHO), female infertility is defined when the female reproductive system is unable to secure a clinical pregnancy after 12 months or more of regular unprotected sexual intercourse [1,2]. Furthermore, infertility is a multifactorial disorder and, therefore, a clinical challenge [3,4]. The prevalence of infertility in reproductive-aged couples is increasing, and nowadays, infertility is estimated to affect more than 186 million people (8% to 12% of couples at the reproductive age) [4]. 

Periodontitis (PD) is a chronic periodontal disease characterized by inflamed gums and bone destruction surrounding the teeth due to a dysbiotic microflora that induces the upregulation of inflammatory mediators [5,6]. The systemic repercussions caused by PD are well established [7,8,9,10,11], and PD has been consistently associated with several chronic diseases [12,13,14,15,16,17,18,19]. Furthermore, PD negatively impacts a patient’s oral health-related quality of life (OHRQoL) [20], though it can be restored after periodontal therapy [21].

Recently, several possible associations were proposed to explain how PD acts as a modifiable risk factor in female infertility related conditions [22,23,24,25,26,27,28,29,30,31,32]. Nevertheless, to the best of our knowledge, there is a lack of studies concerning the periodontal status of women seeking fertility treatment. The understanding on periodontal status, hygiene habits and impact on OHRQoL might be of great scientific and clinical interest.

Therefore, our primary aim was to compare the periodontal status of females referenced to a fertility clinic with matched females from a representative epidemiological sample. Our secondary aims were to investigate if periodontal clinical measures differ between these two groups of females and how they impact OHRQoL. Our null hypothesis is that females referenced to a fertility clinic have similar periodontal status to matched females from a representative epidemiological sample.

## 2. Materials and Methods

### 2.1. Study Design and Participants

Eighteen consecutive female patients referenced for fertility treatment at the Center for Infertility and Medically Assisted Reproduction (CIMAR) at Garcia de Orta Hospital (Almada, Portugal), were enrolled in this cross-sectional study, between February and March 2020. Informed consent was obtained from all women prior to commencement. The exclusion criteria for this study group, labeled as the Fertility Clinic Group (FCG), were as follows: less than 18 years of age; systemic antibiotics, corticosteroids and/or immunosuppressive drugs within the last 3 months prior to periodontal examination or periodontal treatment within the last year. The control group (CG) consisted of 18 fertile female participants, matched for age, race and BMI, randomly selected from the Study of Periodontal Health in Almada-Seixal (SoPHiAS) sample [33]. 

The present cross-sectional study was approved by each institutional review board, including the Research Ethics Committee of the Garcia de Orta Hospital Investigation Center EPE (Portugal) (Approval number: Process: 90/2019) and the Research Ethics Committee of the Regional Health Administration of Lisbon and Tagus Valley, IP (Portugal) (Approval number: 3525/CES/2018). The study was performed following the STrengthening the Reporting of OBservational studies in Epidemiology (STROBE) guidelines [34].( Supplementary Materials)

### 2.2. Periodontal Assessment

Full-mouth periodontal examination was conducted by calibrated investigators (VM and JM) under proper lighting with the individuals seated on a regular adjustable stretcher. All fully erupted teeth, excluding third molars, implants and retained roots, were examined circumferential at six sites per tooth (mesiobuccal, buccal, distobuccal, mesiolingual, lingual and distolingual). The following parameters were measured: the number of missing teeth, plaque index (PI) [35] and bleeding on probing (BoP) [36] were recorded as present or absent; gingival recession (REC), probing pocket depth (PPD) and clinical attachment loss (CAL) were measured using a manual periodontal 15 UNC probe (Hu-Friedy^®^ Manufacturing Inc., Chicago, IL, USA); furcation involvement (FI) was assessed using a Nabers probe (2N Hu-Friedy, Chicago, IL, USA) [37]; and tooth mobility was appraised following Miller [38]. 

According to the European Federation of Periodontology/American Association of Periodontology (EFP/AAP) 2017 case definitions, gingivitis case (GC) was defined in intact or reduced periodontium [39]. Periodontitis cases were defined as detectable interdental CAL at ≥ 2 non-adjacent teeth; or buccal or oral CAL ≥ 3 mm with PPD > 3 mm detectable at ≥ 2 teeth, and the observed CAL was not attributed to non-periodontal causes [40]. The periodontitis severity stage was defined as per interdental CAL at the site of the greatest loss of 1–2 mm, 3–4 and ≥ 5 was considered as mild (Stage I), moderate (Stage II) and severe (Stage III and Stage IV), respectively [40]. Extent and distribution were defined as localized, if < 30% of teeth were involved, or generalized, if ≥ 30% of teeth were involved [40].

Additionally, the periodontal inflamed surface area (PISA) and the periodontal epithelial surface area (PESA) were calculated to depict the pocket epithelial surface area of bleeding and the root surface area in a particular tooth (in mm^2^), respectively [41].

### 2.3. Measurement Reliability and Reproducibility

Two examiners (VM and JB) were trained prior to periodontal data collection. A total of 10 volunteers seeking dental care at Egas Moniz Dental Clinic (EMDC) were randomly selected. Periodontal measurements were collected and repeated one week later. These patients were not further involved in the study. Measurement reliability and reproducibility were assessed by the intraclass correlation coefficient (ICC). The intra-examiner ICC ranged from 0.97 to 0.99, for both PPD and CAL.

### 2.4. Sociodemographic and Medical Questionnaires

By means of a structured questionnaire, the following information was collected: (1) gender, age, educational level; (2) smoking habits; (3) oral hygiene-related behaviors (toothbrushing frequency and interproximal cleaning); (5) attitudes and awareness towards oral health; (6) presence of systemic diseases (such as diabetes mellitus and hypertension).

Education levels were categorically registered according to the 2011 International Standard Classification of Education (ISCED-2011): Elementary (ISCED 1–2 levels), Middle (ISCED 3–4 levels), Higher (ISCED 5–8 levels) [42]. Smoking status was defined as non-smoker (category 0), former smoker (category 1) or active smoker (category 2).

### 2.5. OHRQoL Questionnaire

Before the periodontal examination, we applied the Portuguese versions of the Oral Health Impact Profile-14 (OHIP-14-PT) to assess self-perceived OHRQoL [43]. OHIP-14-PT is a 14-question tool, containing seven domains: functional limitation, physical pain, psychological discomfort, physical disability, psychological disability, social disability and handicap of OHRQoL. Each question is scored categorically (0 = never, 1 = hardly ever, 2 = occasionally, 3 = fairly often and 4 = very often). A higher score indicates poorer OHRQoL [44]. 

### 2.6. Statistical Analysis

Data analyses were conducted for all participants and for sample subsets, according to the defined groups, FCG and CG. The OHIP-14 scores were calculated and their correspondent descriptive measures, mean and standard deviation (SD), were computed. For the purpose of analysis, the OHIP-14 score was handled as a continuous variable. Chi-square and Mann–Whitney tests were used to compare categorical and continuous variables between the groups. All statistical analyses were performed in R version 4.0.0 (R Foundation for Statistical Computing, Vienna, Austria). A level of significance of 5% was considered in all inferential analyses.

## 3. Results

### 3.1. Characteristics of Included Studies

The patient characteristics from both groups, FCG and CG, are listed in Table 1. There were no differences between sociodemographic, anthropometric and oral hygiene habits characteristics. In both groups, females had middle to higher education. Most of them reported a daily toothbrushing frequency of two to three times and no interdental cleaning habits. Regarding smoking habits, the CG presented more active smokers than the FCG, however, without statistical significance.

### 3.2. Prevalence of Periodontitis and Periodontal Clinical Measures

Women from the FCG presented a higher occurrence of PD than in the CG (Table 2). Overall, half of the population in the FCG presented with periodontal disease, with one third of the total having PD (33.3%). In the CG, the prevalence of periodontal disease, (33.3%) and PD (22.2%), respectively, were lower. Additionally, while the FCG presented both the localized and generalized extent of PPD, the CG only showed localized PD cases.

Then, we compared the periodontal parameters of the women in the FCG and CG (Table 2). FCG participants presented significantly higher values of PESA, PPD, CAL and number of missing teeth than control females (*p* < 0.05).

### 3.3. OHRQoL

Next, we compared self-perceived OHRQoL of the FCG and CG (Table 3). FCG participants expressed better OHRQoL than the controls (*p* < 0.001). Of which, Physical pain, Psychological discomfort and Psychological disability domains were found to be higher in the CG.

## 4. Discussion

The results of this study show that women seeking fertility treatment presented higher levels of periodontitis compared to matched females from a representative epidemiological sample. Comprehensively, the present findings might contribute to learning that PD may be more frequent in women seeking infertility therapy than women in the general population. Furthermore, this particular population had more severe PD and significantly worse periodontal clinical characteristics such as PESA, PPD and CAL. However, as a pilot study, our results give strong evidence that a larger study should be undertaken to confirm such results.

Furthermore, FCG women presented worse periodontal measures according to age, mainly over 30 years old. On the one hand, these outcomes are in line with evidence from the SoPHiAS survey [33] and other studies in the Portuguese population [45,46], where age was a key risk factor [33]. Additionally, age is a ubiquitous risk factor for fertility. This finding has wide implications since this group of women was seeking a successful pregnancy. Growing evidence supports a relationship between PD and female infertility related conditions, namely polycystic ovary syndrome (PCOS) [22,23,25,26,27,28,47,48,49,50,51], endometriosis [29,30] and obesity [52,53,54,55,56]. Furthermore, recent studies have substantiated the relationship between maternal PD as a potential risk factor of adverse pregnancy outcomes [57,58,59,60,61,62,63,64]. Periodontal therapy may prevent or reduce perinatal and maternal morbidity and mortality, especially in mothers at high risk [65,66], though a Cochrane review reported unclear evidence that periodontal treatment during pregnancy has an impact on preterm birth [67]. However, it should be noted that the discrepancy of periodontal case definitions influence the association of PD with adverse pregnancy outcomes [61]. Overall, we anticipate that periodontal appointments might be important for prenatal care to possibly increase fertility success and avoid adverse pregnancy outcomes.

Additionally, PD has been linked to other factors such as oral health behaviors and psychological characteristics [20,68,69]. Nevertheless, no studies have assessed OHRQoL, periodontal status and oral hygiene habits among women attending a fertility clinic. Interestingly, FCG women reported less OHRQoL influence than the CG, though larger samples are warranted to confirm this difference. In this context, our results expand on previous evidence that OHRQoL reflects oral symptoms and functional limitations, and also its impact on the psychosocial status of the self [70]. Thus, oral health self-perception is influenced by the individual’s experiences, perceptions, expectations and ability to adapt to different adverse circumstances [71]. In this sense, our data suggest that women seeking fertility care might disregard the impact of oral health, and for instance periodontal disease, since they may have other priorities and concerns, yet this warrants further confirmation. However, the least concern was already described in pregnant women, where they devalued the periodontal state in terms of its OHRQoL [72], although there is contrary evidence [73]. Insofar, these data have no parallel and may suggest a lack of awareness in this group of interest, although this should be investigated in more detail in future studies.

Currently, the proposed biological mechanisms linking PD and infertility are based on the spread of periodontal bacteria and their products into the bloodstream, with local and systemic inflammatory burden, oxidative stress and leukocyte response [22,23,74,75]. Comprehensively, a diseased periodontium in women leads to a subclinical chronic inflammatory status [7,14,76,77,78,79] that potentiates an oxidative stress environment, and therefore impacts the endocrine system and decreases fertility [25,26]. Moreover, several female infertility associated conditions (such as PCOS, endometriosis and obesity) were implicated with PD [23,24,28,29,30,51,52,54,76,80,81,82,83].

The present study had strengths and limitations. The most important limitation is the small number of enrolled subjects. Furthermore, since women were evaluated at the first appointment at the infertility clinic, we did not collect the infertility etiology of each woman. However, patients in the FCG, included consecutively, were representative of a nationwide reference fertility center. In addition, the control group was randomly selected from a representative sample within the same geographic area of the fertility center and matched for age, race and BMI characteristics. This cross-sectional study precludes any inference of causality or time-dependent link between the analyzed variables. Hence, future prospective studies shall consider the influence of PD onset and progression on the fertility clinical status of these particular patients. Nevertheless, this is the first study to compare the periodontal status of women seeking care at a fertility clinic with other women. We employed appropriate periodontal assessment methods with the current PD case definition and its studied upsides [40,84,85], and the measures of interest were assessed by calibrated examiners. Therefore, in our view, these results are up-to-date and of relevant scientific interest.

## 5. Conclusions

Within the limitation of this study, females referenced for fertility treatment presented worse periodontal measures than females from a representative control sample. These preliminary results may support future prospective studies to further explore the periodontal status and possible consequences in women seeking fertility care.

## Figures and Tables

**Table 1:** Participant characteristics.

	FCG (*n* = 18)	CG (*n* = 18)	*p*-Value *
**Age, mean (SD) (years)**	33.9 (4.3)	34.1 (3.8)	0.914
**BMI, mean (SD)**	24.5 (3.9)	23.1 (3.7)	0.279
**Race, *n* (%)**			
Black	5 (27.8)	5 (27.8)	1.000
White	13 (72.2)	13 (72.2)	
**Smoking, *n* (%)**			
No	15 (83.3)	11 (61.1)	0.270
Former	1 (5.6)	1 (5.6)	
Active	2 (11.1)	6 (33.3)	
**Education, *n* (%)**			
Middle	10 (55.6)	13 (72.2)	0.488
Higher	8 (44.4)	5 (27.8)	
**Toothbrushing daily frequency, *n* (%)**			
1 or less	3 (16.7)	3 (16.7)	0.790
2 or more	15 (83.3)	15 (83.3)	
**Interdental cleaning, *n* (%)**			
Yes	9 (50.0)	8 (44.4)	0.117
No/Never	9 (50.0)	10 (55.6)	
**Last dental visit, *n* (%)**			
<6 months	6 (33.3)	4 (22.2)	0.176
6 to 12 months	7 (38.9)	4 (22.2)	
>12 months	5 (27.8)	10 (55.6)	
**Denture, *n* (%)**	1 (5.6)	0 (0.0)	-
**Systemic conditions, *n* (%)**			
Diabetes Mellitus	0 (0.0)	0 (0.0)	-
Hypertension	1 (5.6)	0 (0.0)	-

* Chi-square test for categorical variables, Mann–Whitney test for continuous variables.

**Table 2:** Periodontal status and clinical measures for the FCG and CG.

	FCG (*n* = 18)	CG (*n* = 18)	*p*-Value *
**Periodontal status, *n* (%)**			
Healthy	9 (50.0)	12 (66.7)	-
Gingivitis	3 (16.7)	2 (11.1)	
Periodontitis	6 (33.3)	4 (22.2)	
**Periodontitis staging, *n* (%)**	*n* = 6	*n* = 4	
Mild (Stage I)	3 (16.7)	3 (16.7)	-
Moderate (Stage II)	2 (11.1)	1 (5.6)	
Severe (Stage III)	1 (5.6)	0 (0)	
**Periodontitis extent, *n* (%)**	*n* = 6	*n* = 4	
Localized	5 (83.3)	4 (100.0)	-
Generalized	1 (16.7)	0 (0.0)	
PI, mean (SD) (%)	0.32 (0.98)	4.66 (13.55)	0.103
Calculus, mean (SD) (%)	2.69 (4.93)	4.64 (7.07)	0.203
BoP, mean (SD) (%)	18.67 (29)	6.53 (9.94)	0.156
PISA, mean (SD) (mm^2^)	76.27 (122.62)	32.77 (50.77)	0.271
PESA, mean (SD) (mm^2^)	295.61 (69.82)	250.48 (74.59)	0.020
PPD, mean (SD) (mm)	2.07 (0.48)	1.63 (0.39)	0.010
CAL, mean (SD) (mm)	2.10 (0.48)	1.72 (0.40)	0.007
N° of missing teeth, mean (SD)	0.8 (1.0)	1.7 (2.1)	<0.001

* Mann–Whitney test.

**Table 3:** OHRQoL in both groups, FCG and CG.

	FCG (*n* = 18)	CG (*n* = 18)	*p*-Value *
OHIP-14, Mean (SD)	2.4 (1.7)	9.6 (13.8)	<0.001
OHIP-14 Domains, Mean (SD)			
Functional Limitation	0.0 (0.0)	0.1 (1.5)	0.055
**Physical Pain**	**2.2 (1.9)**	**2.4 (2.9)**	**<0.001**
**Psychological Discomfort**	**0.3 (0.9)**	**1.8 (2.8)**	**0.009**
Physical Disability	0.0 (0.0)	1.5 (2.9)	0.058
**Psychological Disability**	**0.0 (0.0)**	**1.6 (2.3)**	**0.009**
Social Disability	0.0 (0.0)	0.7 (1.6)	0.058
Handicap	0.0 (0.0)	0.9 (2.1)	0.055

* Mann–Whitney test, bold is for *p*<0.05

## Supplementary Materials

The following are available online at https://www.mdpi.com/1660-4601/17/15/5281/s1.
